# *APOE ε*4 carrier status moderates the effect of lifestyle factors on cognitive reserve

**DOI:** 10.1002/alz.14304

**Published:** 2024-10-11

**Authors:** Deirdre M. O'Shea, Andrea S. Zhang, Katana Rader, Rebecca L. Shakour, Lilah Besser, James E. Galvin

**Affiliations:** ^1^ Comprehensive Center for Brain Health Department of Neurology University of Miami Miller School of Medicine Boca Raton Florida USA; ^2^ University of Miami Miller School of Medicine Miami Florida USA

**Keywords:** Alzheimer's disease, *APOE ε*4 status, cognitive function, cognitive reserve, lifestyle factors

## Abstract

**INTRODUCTION:**

This study examines the role of lifestyle factors in cognitive reserve among older adults, focusing on the moderating effect of apolipoprotein E (*APOE*) *ε*4 status.

**METHODS:**

Data from 157 participants aged 45 and older from the Healthy Brain Initiative (HBI) were analyzed. Cognitive reserve was estimated using residual scores from Cognivue Clarity tests after accounting for brain atrophy and white matter hyperintensities (WMHs). Lifestyle factors included education, occupational attainment, physical activity, social engagement, diet, and mindfulness. Structural equation models were conducted to assess interactions.

**RESULTS:**

Significant interactions were found between *APOE ε*4 status and mindfulness and social engagement on cognitive reserve, indicating stronger associations for *APOE ε*4 carriers.

**DISCUSSION:**

*APOE ε*4 carriers may benefit more from certain lifestyle factors, potentially through stress reduction and anti‐inflammatory pathways. These findings support integrating *APOE ε*4 genetic screening into personalized prevention strategies to enhance interventions aimed at preserving cognitive function and delaying dementia onset in at‐risk populations.

**Highlights:**

- Mindfulness and social engagement have increased cognitive reserve in *APOE ε*4 carriers.
- Study uses residual scores from Cognivue Clarity tests to estimate cognitive reserve.
- *APOE ε*4 carriers show stronger associations with certain lifestyle factors on cognitive reserve.
- Personalized interventions could enhance cognitive resilience in genetically at‐risk populations.
- Comprehensive assessment of multiple lifestyle factors highlights targeted intervention benefits.

## 1 BACKGROUND

Cognitive reserve has emerged as a core framework for understanding heterogeneity in cognitive aging^1^, ^2^ and the onset and progression of dementia, including Alzheimer's disease and related dementias (ADRD).^3^, ^4^ It refers to the capacity to maintain cognitive function despite neuropathological damage, a concept used to explain heterogeneity in clinical outcomes among individuals with similar levels of brain pathology.^5^ Certain modifiable factors, such as educational and occupational attainment, are thought to increase cognitive reserve and are often used as proxy measures of cognitive reserve.^6^, ^7^ Several other modifiable lifestyle factors, such as physical activity, healthy diet, and social and cognitive leisure activities, have been associated with better brain and cognitive aging and reduced risk for dementia.^8^, ^9^, ^10^ According to the 2020 Lancet Commission Report, 12 modifiable factors, including low educational attainment, social isolation, and several cardiovascular‐related factors, account for approximately 40% of dementia cases.^11^ These factors are posited to increase dementia risk potentially through their cumulative adverse effects on brain health and function.^12^ Despite the link between lifestyle factors and cognitive reserve, a recent review highlighted a lack of direct research on how factors like physical activity, diet, and practices such as meditation and mindfulness specifically contribute to cognitive reserve.^13^

Evidence suggests that genetic factors may influence the impact of lifestyle factors on cognitive health^14^, ^15^, ^16^; specifically, the apolipoprotein E (*APOE*) *ε*4 allele is a well‐established genetic risk factor for Alzheimer's disease.^17^, ^18^ The *APOE ε*4 allele has been associated with a higher accumulation of amyloid plaques and neurofibrillary tangles,^19^ hallmark features of Alzheimer's disease, which contribute to accelerated cognitive decline. Studies have demonstrated that the cognitive benefits associated with various modifiable factors, such as education, physical activity, and diet, may be more pronounced in *APOE ε*4 carriers compared to noncarriers.^14^, ^20^, ^21^ Neuroimaging findings further support these findings. For instance, greater physical activity has been linked to increased hippocampal volumes, with the strongest effects observed in *APOE ε*4 carriers.^22^, ^23^

While some modifiable factors are known to benefit cognition, a recent review highlighted a substantial gap in the literature regarding their specific contributions to cognitive reserve,^13^ particularly in the context of genetic risk. For instance, one study found that physical and cognitive activities were generally linked to greater cognitive speed reserve, but these benefits were reduced in women who were *APOE ε*4 carriers.^24^ However, this study only considered a limited range of lifestyle factors (physical and cognitive activity) and assessed the moderating effect of *APOE ε*4 within sex‐stratified analyses, which may obscure its broader impact. Moreover, most research has focused on individual lifestyle factors rather than their combined effects, overlooking potential synergistic interactions that could enhance cognitive reserve in individuals with genetic risk.

In this study, cognitive reserve is operationalized as the residual variance in cognitive performance after accounting for brain atrophy and white matter hyperintensities (WMHs). This method isolates cognitive function not directly related to structural brain changes, providing a more precise measure of cognitive reserve. Lifestyle factors like education and occupation are often used as proxies for cognitive reserve rather than direct measures. By examining these factors about the cognitive reserve residual, we aim to better understand how various lifestyle practices contribute to cognitive reserve, especially in the context of genetic risk, such as *APOE ε*4 status.

The current study had two main aims: (1) to examine the combined and specific associations between various lifestyle factors (eg, education, occupational attainment, physical activity, leisure activities, social engagement, diet, and mindfulness) and cognitive reserve, defined as residual variance in cognition after accounting for brain imaging variables, in a community‐based sample of older adults without dementia; and (2) to explore whether these associations differed by *APOE ε*4 carrier status, given its link to increased cognitive decline risk. We hypothesized stronger positive effects of both well‐established (physical activity) and less studied (mindfulness) lifestyle factors in *APOE ε*4 carriers, based on prior findings.

## 2 METHODS

### 2.1 Participants

Data for the current study were obtained from baseline visits of participants enrolled in the Healthy Brain Initiative (HBI). Full details of the HBI study procedures are reported elsewhere.^25^ In brief, participants are recruited using community‐based events, physician referrals, and flyers. Participants are followed annually and undergo comprehensive cognitive, physical, functional, behavioral, and neurological examinations at baseline and each subsequent annual visit. Whole blood and plasma samples are collected at each visit. Neuroimaging is collected at baseline and every other year. Participants provide full informed consent before they participate in the study. The study is approved by the University of Miami Institutional Review Board. Inclusion criteria for participation require the participant to be 45 or older, have a study partner, not have a prior diagnosis of dementia, and be eligible to undergo magnetic resonance imaging (MRI). We included data from participants aged 45 years and older to capture a broad range of midlife to older adults, as prior research indicates that cognitive decline and the influence of genetic factors like *APOE ε*4 may start to manifest in midlife.^26^, ^27^ The final dataset for the current study included 157 participants with complete baseline cognitive, psychosocial, *APOE ε*4 genotype, and structural neuroimaging data.

### 2.2 Measures

#### 2.2.1 Global cognition

Global cognition was assessed using Cognivue Clarity, a Food and Drug Administration (FDA)‐cleared computerized test that aids in the evaluation of cognitive decline or dementia in patients aged 55 to 95 years.^28^ Cognivue Clarity takes approximately 10 minutes to administer. It comprises an automated sequence of subtests that assess the following cognitive domains: visuomotor, visual attention, language, executive functioning, processing speed, learning, and memory processing. Total scores are estimated based on the average performance across these domains. Total scores range from 0 to 100 and were used in the current study to estimate participants' cognitive reserve (described below). Higher scores represent better cognitive function.

#### 2.2.2 Cognitive reserve

Cognitive reserve was estimated by regressing Cognivue performances on total atrophy scores and WMH volumes. The resulting standardized residual represents the variance not explained by these brain variables in cognitive performances, where higher scores represent higher cognitive reserve. This is an approach that has been widely used to assess cognitive reserve, whether in specific cognitive domains or across general cognitive ability.^29^, ^30^ Although some studies regress the effect of demographic variables (ie, age and sex), these variables were included as covariates within this sample in the primary analysis instead. Higher total scores indicate better cognitive performance.

#### 2.2.3 *APOE ε*4 carrier status

ApoE proteotyping (identical to genotyping) was performed using whole blood samples collected at participants' baseline visits that were initially stored onsite and frozen at −80°C before shipping to C2N Diagnostics (St. Louis, MO). ApoE proteotyping was conducted by C2N Diagnostics using a method that identifies the presence or absence of particular apoE isoform‐specific peptides through ratio dot product (rdotp) scores provided by Skyline.^31^ Peptides with rdotp values of 0.99 or higher were considered present, while those below 0.99 were considered absent, and an in‐house R script was used to generate the genotypes based on these results.^32^ ApoE proteotypes in our sample included 2/2 (*n* = 1), 2/3 (*n* = 12), 2/4 (*n* = 4), 3/3 (*n* = 97), 3/4 (*n* = 41), and 4/4 (*n* = 2). For the current study, apoE proteotypes were dichotomized into carriers, defined as having at least one *ε*4 allele (coded as 1, n = 36), including those who were *ε*2/*ε*4 carriers, versus noncarriers, designated as individuals without the *ε*4 allele (coded as 0, *n* = 82). Although some studies have excluded *ε*2/*ε*4 carriers due to the potential protective and mitigating effect of the *ε*2 allele on the *ε*4 allele,^33^ other studies have not.^34^ Given that our sample included only four individuals with this genotype and therefore determined to have a minimal effect on the results, we included these individuals and classified them as *ε*4 carriers.

**RESEARCH IN CONTEXT**

1. **Systematic review**: The authors reviewed the literature using databases like PubMed, focusing on studies linking lifestyle factors, cognitive reserve, and *APOE ε*4 status. Search terms included “cognitive reserve,” “lifestyle factors,” “*APOE ε*4,” “physical activity,” “diet,” “mindfulness,” and “social engagement.” This review identified research gaps directly connecting these factors to cognitive reserve in the context of *APOE ε*4.
2. **Interpretation**: Our findings show that mindfulness and social engagement significantly enhance cognitive reserve in *APOE ε*4 carriers, highlighting the potential of personalized lifestyle interventions to mitigate genetic risk factors for cognitive decline.
3. **Future directions**: Future research should explore longitudinal effects of lifestyle changes on cognitive reserve in *APOE ε*4 carriers, investigate the neurobiological mechanisms involved, and develop targeted interventions to optimize cognitive health in genetically at‐risk populations.

#### 2.2.4 Lifestyle activities

Data on lifestyle factors were derived from the subscales of the Resilience Index (RI).^35^ The RI is a previously validated composite measure of six previously validated subscales that include the total scores from the Cognitive Reserve Unit Scale (CRUS),^36^ the Social Engagement Scale,^37^ the Quick Physical Activity Rating,^38^ the Cognitive & Leisure Activity Scale,^37^ the Mediterranean‐DASH Intervention for Neurodegenerative Delay (MIND) diet score,^39^ and the Applied Mindfulness Process Scale (AMPS).^40^ Scores on RI range from 1 to 378, with higher scores indicating greater resilience and better brain health. A detailed description of each subscale is presented in Table S1 in the Supplementary File.

### 2.3 MRI protocol

All participants underwent structural brain MRI scans using a GE 3 Tesla 750 W scanner. The MRI protocol included T1‐weighted structural images, T2‐weighted images, and fluid‐attenuated inversion recovery (FLAIR) images. High‐resolution 3D sagittal images were obtained with magnetization‐prepared rapid gradient‐echo (MPRAGE) sequences, followed by axial and coronal reconstructions. Multiple corrections were applied to the raw MRI images to ensure quality. These included adjustments for geometric distortion due to gradient nonlinearity, field inhomogeneity correction, histogram‐peak sharpening, and site‐specific scanner features and field strength adjustments.

#### 2.3.1 Brain MRI volumes

Cortical volumes were measured using the Combinostics cNeuro suite (Tampere, Finland), an FDA‐approved quantitative analysis tool. This suite employs artificial intelligence (AI) technology to enhance the imaging analysis speed, accuracy, precision, and robustness, thus improving the quality of research data. The AI‐driven Combinostics cMRI suite has shown performance comparable to manual segmentation, with Pearson correlation coefficients ranging from 0.84 to 0.99.^41^

Measures of cortical atrophy and WMH were generated using the cNeuro suite. The cortical atrophy score (CAS) is a z‐score estimate of global cortical atrophy developed using the widely used Global Cortical Atrophy visual rating scale. The CAS is adjusted for age, sex, and head size (a proxy for intracranial volume), based on a reference sample of individuals aged 50 to 90. Research shows that the CAS outperforms total cerebral cortex volume measures (area under the curve [AUC] = 0.823) and combined cerebral gray and white matter volume (AUC = 0.833) in distinguishing early‐stage Alzheimer's disease from cognitively normal (CN) individuals, with an AUC of 0.873.^41^ Higher scores indicate greater global cortical atrophy. Total brain tissue WMH was also extracted using the cNeuro suite, with higher scores indicating more WMHs (adjusted for age, sex, and head size). Cortical atrophy and WMHs were selected as the primary MRI measures to estimate global changes due to aging, ADRD, and lifestyle, and as a marker relevant to cognitive function.^42^ Given the WMH variable was highly skewed and kurtotic, a Box‐Cox transformation was applied (following minimal improvement after a log transformation), which effectively reduced skewness and kurtosis, resulting in a normalized distribution.

### 2.4 Cognitive status

The cognitive status of each participant was determined by an initial “research” diagnosis of CN, mild cognitive impairment (MCI), or dementia. These diagnoses were established through a consensus conference involving a neurologist, neuropsychologist, and nurse practitioners. The cognitive assessment included a comprehensive battery of neuropsychological tests that evaluated multiple cognitive domains, such as memory, executive function, language, visuospatial abilities, and attention. The cognitive test measures administered to participants and considered in the diagnoses were derived from the National Alzheimer's Coordinating Center (NACC) Uniform Data Set (UDS version 3).^43^ Specific measures included are described in the Supplementary File. Cognitive performances (excluding Cognivue), medical history, and Clinical Dementia Rating scores (ie, a global score of 0.5 indicative of MCI) were used to inform diagnostic classifications by the National Institute on Aging–Alzheimer's Association (NIA‐AA) clinical criteria.^44^ Among the participants in the current study, 50 individuals were classified as having MCI, 107 were classified as CN, and no individuals were diagnosed with dementia.

### 2.5 Covariates

Sex/gender (males, coded as 1, females coded as 2), age (years), and years of education were calculated based on self‐reported levels of education.

### 2.6 Statistical analysis

Descriptive analyses were conducted in SPSS Statistics (IBM Corp., Released 2021; IBM SPSS Statistics for Windows, Version 28.0; Armonk, NY: IBM Corp.). All other analyses were conducted in R studio (version 4.3.2). Figures were generated with the ggplot package in R studio.^45^ Differences in participants' variables by sex and *APOE ε*4 carrier status were assessed using Mann–Whitney *U* tests, given its robustness with non‐normally distributed data and unequal sample sizes and chi‐square tests for categorical variables.

**FIGURE 1:**
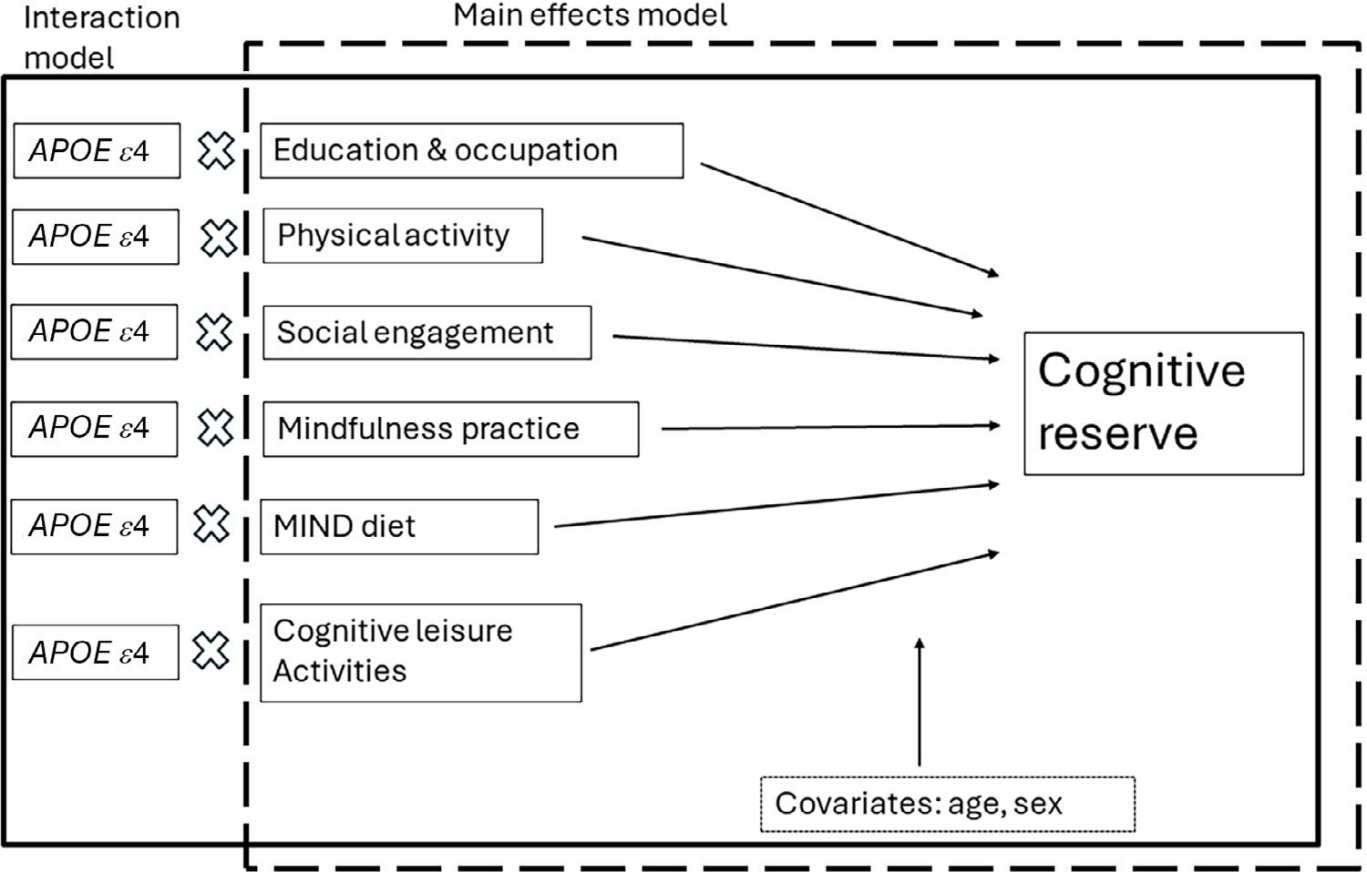
Conceptual SEM models for main and interaction effects of lifestyle factors on cognitive reserve. *APOE*, apolipoprotein E; MIND, Mediterranean‐DASH Intervention for Neurodegenerative Delay; SEM, structural equation model.

A structural equation model (SEM) was employed to examine the direct effects of each of the lifestyle factors on the cognitive reserve residual while controlling for age and sex. The lifestyle factors included education and occupation (ie, CRUS), social engagement, physical activity, cognitive leisure activity, MIND diet, and mindfulness practice. Given the anticipated correlations among these predictors, covariances between all pairs of lifestyle factors were specified in the model. The SEM was estimated using the maximum likelihood (ML) method with the NLMINB optimization algorithm in the lavaan package in R. The model's goodness‐of‐fit was evaluated using the chi‐square test, where a nonsignificant result indicates a good fit to the data. The standardized regression coefficients (β) were reported to assess the direct effects of each predictor on the dependent variable, cognitive reserve residual. Standard errors and *p*‐values were provided for all estimates to determine the significance of the effects. The SEM was rerun to include interaction terms between *APOE ε*4 status and each of the lifestyle factors. Figure 1 illustrates the conceptual models for the SEM analyses. Simple slope analyses were conducted to further explore significant interaction terms. All variables were converted to z‐scores based on the current study sample's mean and standard deviation (SD) before analyses to facilitate interpretation. All statistical analyses were conducted with a two‐tailed significance level of 𝛼 = 0.05. Effect sizes were interpreted using standardized coefficients. To assess the study's power for detecting interaction effects in a linear regression model, a power analysis was conducted using Cohen's *f* ^2^ effect sizes: 0.02 (small), 0.15 (medium), and 0.35 (large). With a sample size of 157, an alpha of 0.05, and a desired power of 0.80, the analysis confirmed sufficient power to detect medium and large effects but not small effects. We also employed adjustments for multiple comparisons using the false discovery rate (FDR) (ie, the Benjamini–Hochberg method) to control for Type I error across the multiple analyses conducted.

### 2.7 Sensitivity analysis

To ensure that our findings were not influenced by the age distribution of our sample, we conducted a stratified analysis by age group (< 65 years [*n* = 43] and ≥65 years [*n* = 114]). To assess the independent contribution of education and occupation (versus using them in a combined scale, ie, the CRUS), we reran the SEM interaction model with these as separate factors. To examine whether cognitive status (ie, normal vs MCI) influenced associations between *APOE ε*4 carrier status and lifestyle factors on cognitive reserve, we conducted an exploratory SEM analysis to investigate the three‐way interactions between *APOE ε*4 carrier status, lifestyle factors, and cognitive status, controlling for age and sex.

## 3 RESULTS

### 3.1 Descriptive statistics

Sample characteristics are summarized in Table 1. The mean (± SD) age was 69.22 ± 9.36 years, mean education years = 16.38 (2.56), ≈66% were women, 86% non‐Hispanic White, 9% non‐Hispanic Black, 1.5% Asian, 2% Hispanic and 1.5% other race/ethnicity. Results from Mann–Whitney *U* tests revealed significant differences between *APOE ε*4 carriers and noncarriers in age (carrier status: *U* = 1818.00, *Z* = −2.942, *p* = 0.003), with noncarriers being older on average. There were no significant differences between carrier status and any of the lifestyle variables or the cognitive reserve residual (all *p* > 0.05). Comparisons of men and women revealed that men had higher mean ranks on the CRUS scale compared to females (*U* = 1743.50, *Z* = −3.708, *p* < 0.001). No significant sex differences were found for the other measures (all *p* > 0.05). Chi‐square tests showed no statistically significant association between sex and *APOE* genotype, χ^2^(1, 157) = 0.03, *p* = 0.873, or between *APOE ε*4 carrier status and cognitive diagnosis, χ^2^(1, 157) = 0.00, *p* = 0.990. Similarly, the chi‐square test did not reveal a statistically significant association between gender and cognitive status, χ^2^(1, 157) = 0.79, *p* = 0.375.

**TABLE 1:** Characteristics of the study sample (whole sample and by *APOE ε*4 carrier status).

Whole sample	Minimum	Maximum	Mean	SD
Age	45	92	69.22	9.36
Education (years)	9	20	16.38	2.56
RI	115	283.5	180.03	30.14
CRUS	19	66	51.31	11.22
Social engagement	2	4	3.27	0.64
QPAR	5	129	38.38	20.83
CLAS	10	53	32.49	8.56
MIND	5	14.5	10.21	2.07
AMPS	18	60	44.38	8.64
Cognitive reserve	−3.77	1.79	0	0.99
MCI, *n* (%)	50 (31.8)			
Female, *n* (%)	104 (66.2)			
*APOE ε*4 carriers	47 (29.9)			
***APOE ε*4 noncarriers, *n* = 110**				
Age	46	92	70.58	9.23
Education (years)	9	20	16.28	2.69
RI	115	283.5	179.55	31.53
CRUS	19	66	51.16	11.54
Social engagement	2	4	3.29	0.59
QPAR	5	129	38.24	22.10
CLAS	10	53	31.83	8.94
MIND	5	13.5	10.13	2.10
AMPS	18	60	44.90	8.98
Cognitive reserve	−2.20	1.77	−0.02	0.91
MCI, *n* (%)	35 (31.8)			
Female, *n* (%)	73 (66.4)			
***APOE ε*4 carriers, *n* = 47**				
Age	45	83	66.02	8.95
Education (years)	12	20	16.62	2.24
RI	122.5	240.5	181.149	26.88
CRUS	26	66	52.66	10.53
Social engagement	2	4	3.21	0.75
QPAR	7	95	38.7	20.39
CLAS	16	50	34.04	7.55
MIND	5.5	14.5	10.38	2.01
AMPS	21	56	43.15	7.54
Cognitive reserve	−3.33	1.59	0.06	1.17
MCI, *n* (%)	15 (32)			
Female, *n* (%)	31 (66)			

*Note*: 0 = noncarriers, 1 = carriers.

Abbreviations: AMPS, Applied Mindfulness Process Scale; *APOE ε*4, apolipoprotein E *ε*4 allele status; CLAS, Cognitive Leisure Activity Scale; CRUS, Cognitive Reserve Unit Scale (education and occupation combined); MCI, mild cognitive impairment; MIND, Mediterranean‐DASH Intervention for Neurodegenerative Delay; QPAR, Physical Activity Scale; RI, Resiliency Index.

### 3.2 SEM

The SEM provided a good fit to the data, χ^2^(12) = 10.864, *p* = 0.541, comparative fit index (CFI) = 1.00, Tucker–Lewis index (TLI) = 1.03, root mean square error of approximation (RMSEA) = 0.00, 90% confidence interval [CI] [0.00, 0.08], *p* = 0.812, and standardized root mean square residual (SRMR) = 0.04. The full model explained 24.0% of the variance in cognitive reserve (*R* ^2^ = 0.24). Significant covariances were observed between several lifestyle factors, including between social engagement and cognitive leisure activities (*r* = 0.25, *p* = 0.002) and between cognitive leisure activities and mindfulness (*r* = 0.36, *p* < 0.001), indicating substantial interrelationships among these predictors.

Regarding the direct effects, CRUS was found to be a significant positive predictor of cognitive reserve residual, *β* = 0.280, *p* < 0.001 (FDR adjusted = 0.004). However, social engagement, physical activity, cognitive leisure activities, MIND diet, and mindfulness did not significantly predict cognitive reserve, with *p*‐values ranging from 0.172 to 0.945. Results are summarized in Table 2.

**TABLE 2:** Standardized regression weights for lifestyle factors from the SEM predicting cognitive reserve.

Path	*Β*	*SE*	z‐value	*p*‐value	FDR *p*‐adjust
CRUS	0.280	0.072	3.919	<0.001	0.004
Social engagement	0.065	0.075	0.867	0.386	0.744
Physical Activity	0.102	0.075	1.364	0.172	0.459
Cognitive Activity	0.005	0.077	0.069	0.945	0.945
MIND diet	0.006	0.074	0.086	0.931	0.945
Mindfulness	0.055	0.076	0.730	0.465	0.744
Age	−0.369	0.069	−5.351	<0.001	0.004
Sex	−0.018	0.069	−0.266	0.791	0.945
Model R‐square	0.240				
Chi‐square	10.864			0.541	
*df*	12				

Abbreviations: CRUS, Cognitive Reserve Unit Scale; FDR, false discovery rate; MIND, Mediterranean‐DASH Intervention for Neurodegenerative Delay; SEM, Structural Equation Model.

### 3.3 SEM interactions model

**FIGURE 2:**
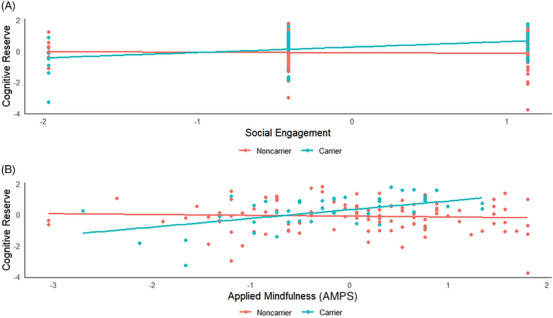
Interaction effects of *APOE ε*4 status and lifestyle factors on cognitive reserve. (A) Interaction between social engagement and *APOE ε*4 status on cognitive reserve, indicating a significant positive effect in *APOE ε*4 carriers. (B) Interaction between applied mindfulness and *APOE ε*4 status, showing a significant positive effect in *APOE ε*4 carriers. AMPS, Applied Mindfulness Process Scale; *APOE*, apolipoprotein E.

The second SEM model, which included interaction terms between lifestyle factors and *APOE ε*4 status, also provided an acceptable fit to the data, χ^2^(54) = 64.96, *p* = 0.146, CFI = 0.914, TLI = 0.866, RMSEA = 0.036, 90% CI [0.000, 0.065], *p* = 0.762, and SRMR = 0.050, and explained 33.8% of the variance in cognitive reserve (*R* ^2^ = 0.338). The inclusion of interaction terms revealed several significant moderating effects of *APOE ε*4 status. Social engagement did not have a significant direct effect, but the interaction with *APOE ε*4 status was significant, *β* = 0.197, *p* = 0.005 (adjusted *p* = 0.020), indicating that social engagement has a stronger effect on cognitive reserve in *APOE ε*4 carriers. Simple slopes analyses further showed that the effect of social engagement on cognitive reserve was significant for *APOE ε*4 carriers (*β* = 0.195, SE = 0.087, *p* = 0.026) but not for noncarriers (*β* = −0.01, SE = 0.028, *p* = 0.818). The interaction between mindfulness and *APOE ε*4 status was significant, *β* = 0.227, *p* = 0.004 (adjusted *p* = 0.020), indicating a stronger effect of the AMPS on cognitive reserve in *APOE ε*4 carriers. Simple slopes analyses showed that the effect of mindfulness on cognitive reserve was significant for *APOE ε*4 carriers (*β* = 0.341, SE = 0.120, *p* = 0.004) but not for noncarriers (*β* = 0.114, SE = 0.073, *p* = 0.120). Results are summarized in Table 3 and significant interaction terms are illustrated in Figure 2.

**TABLE 3:** Regression weights from SEM analysis.

Pathway	*Β*	*SE*	*z*‐value	*p*‐value	FDR *p*‐adjust
CRUS	0.268	0.07	3.972	<0.001	0.012
Social engagement	0.017	0.07	0.24	0.810	0.937
Physical activity	0.057	0.07	0.814	0.416	0.624
Cognitive activity	0.009	0.071	0.124	0.901	0.937
MIND diet	−0.028	0.069	−0.404	0.686	0.915
Mindfulness	0.115	0.071	1.614	0.107	0.214
Age	−0.332	0.067	−4.982	<0.001	
Sex	0.044	0.067	0.652	0.515	
*APOE ε*4	0.114	0.067	1.679	0.093	
*APOE ε*4: CRUS	−0.125	0.075	−1.719	0.086	0.206
*APOE ε*4: Social engagement	0.197	0.064	2.778	0.005	0.020
*APOE ε*4: Physical activity	−0.139	0.075	−1.874	0.061	0.183
*APOE ε*4: Cognitive activity	0.006	0.077	0.079	0.937	0.9370
*APOE ε*4: MIND diet	0.067	0.070	0.956	0.339	0.581
*APOE ε*4: Mindfulness	0.227	0.078	2.893	0.004	0.020
Model R‐square	0.338				
Chi‐square	64.959			0.146	
df	54				

Abbreviations: *APOE*, apolipoprotein E; CRUS, Cognitive Reserve Unit Scale; FDR, false discovery rate; MIND, Mediterranean‐DASH Intervention for Neurodegenerative Delay; SEM, structural equation modeling.

### 3.4 Sensitivity analyses

#### 3.4.1 Age groups

To further explore age group differences, the SEM models with two‐way interaction terms were run separately for younger (less than 65 years) and older (65 years and older) age groups. The models demonstrated that for the Social Engagement × *APOE ε*4 Status term, significant interaction effects were observed in both age groups, with a stronger effect in the younger group (*β* = 0.548, *p* = 0.017) compared to the older group (*β* = 0.195, *p* = 0.047). A significant interaction effect for Mindfulness × *APOE ε*4 status was found in the younger group (*β* = 0.525, *p* = 0.016), while the older group demonstrated a marginally significant interaction for mindfulness (*β* = 0.211, *p* = 0.073). No other significant interactions were found in either age group. The full results of these sensitivity analyses are presented in the Table S2.

#### 3.4.2 Education adjustment

A sensitivity analysis was conducted by separating the CRUS into its components—education and occupation—to examine their independent effects and interactions with *APOE ε*4 status on cognitive reserve. The interaction between *APOE ε*4 status and social engagement (*β* = 0.244, *p* = 0.01, FDR *p*‐adjust = 0.040) and the interaction between *APOE ε*4 status and mindfulness (*β* = 0.232, *p* = 0.008, FDR *p*‐adjust = 0.040) remained significant, similar to the original SEM model. No other significant interactions were observed. The results of these analyses are presented in Table S3.

#### 3.4.3 Three‐way interactions

No significant three‐way interactions were found between *APOE ε*4 status, lifestyle factors, and cognitive status. All three‐way interaction terms had *p*‐values greater than the significance threshold (*p* > 0.05). Results are summarized in Table S4 in the Supplementary File.

## 4 DISCUSSION

Findings from the current study revealed that the positive association between social engagement and mindfulness practice on cognitive reserve was strongest in *APOE ε*4 carriers. In contrast, no such associations were observed for the other subcomponents of the RI, including physical activity, cognitive leisure activities, MIND diets, or cognitive reserve units (education and occupation combined). Additionally, exploratory analyses did not show that these associations varied by cognitive status, although given the small sample sizes of these latter analyses, results should be interpreted with caution.

The *APOE* gene plays a central role in lipid metabolism, neuronal repair, and neuroinflammation and is implicated in the risk of ADRD.^46^, ^47^ Compared to noncarriers of the *ε*4 allele, carriers of one *ε*4 allele exhibit a 2.19‐fold increased risk of developing dementia, while those with two copies of the *ε*4 allele have a 5.97‐fold increased risk of developing dementia.^48^ However, despite the increased vulnerability to dementia and cognitive impairment conferred by the *APOE ε*4 allele, several studies have demonstrated that *APOE ε*4 carriers may benefit more from protective lifestyle factors such as diet^49^ and physical activity.^50^, ^51^ However, it should also be noted that other studies have either not found any effect modification by *APOE ε*4 status^52^, ^53^ or have found the oppositive (ie, that noncarriers benefit more from these lifestyle factors).^54^

The findings from the current study partially corroborate previous research, indicating a more pronounced positive effect of specific lifestyle factors in *APOE ε*4 carriers. Notably, higher scores on social engagement and mindfulness practice were associated with greater cognitive reserve in *APOE ε*4 carriers. In contrast, no significant association between these factors and cognitive reserve was observed in noncarriers. No prior study has investigated the interactive effects of mindfulness‐based practices and *APOE ε*4 carrier status on cognitive outcomes. Nonetheless, mindfulness, similar to cognitive and social activities, has been linked to greater cognitive reserve in older adults, both with and without cognitive impairment^55^, ^56^

There are several potential mechanisms to explain why positive lifestyle factors may benefit *APOE ε*4 carriers more than noncarriers. One possibility is that lifestyle factors could enhance compensatory responses to early neuropathological changes associated with ADRD. This is supported by studies showing that *APOE ε*4 carriers exhibit different brain activity patterns in older adults without dementia, possibly reflecting a compensatory mechanism.^57^, ^58^

Another potential mechanism is that social engagement may attenuate stress responses, which is particularly relevant for *APOE ε*4 carriers who exhibit heightened stress pathways.^59^, ^60^ Reducing stress could enhance cognitive reserve in this genetically vulnerable group, as chronic stress negatively impacts brain health and cognition.^60^, ^61^ Social engagement embodies a multifaceted lifestyle factor encompassing emotional support, cognitive stimulation, and shared experiences, which collectively may contribute to cognitive resilience.^62^ Studies have shown that increased social interactions can provide cognitive challenges that stimulate neural circuits and reduce stress,^63^, ^64^ fostering cognitive reserve and potentially buffering against the stress‐induced cognitive decline seen in *APOE ε*4 carriers.

Similarly, mindfulness practices encapsulate focused attention, awareness, and acceptance and offer a robust framework for stress reduction.^65^ Black and Slavich^66^ demonstrated that mindfulness attenuated inflammatory responses and modulated stress‐related pathways. For *APOE ε*4carriers, who may experience heightened inflammatory states due to their genotype,^67^ mindfulness could offer a direct countermeasure to the allele's pro‐inflammatory tendencies. By promoting neurocognitive resilience through stress reduction and anti‐inflammatory effects, mindfulness may particularly benefit *APOE ε*4 carriers, potentially offsetting their predisposed risk trajectories.

The lack of associations between physical activity, the MIND diet, cognitive leisure activities, and education/occupation (CRUS) in *APOE ε*4 carriers may stem from the multifactorial effects of these activities on the brain, which might not interact directly with *APOE ε*4 status as do mindfulness and social engagement. Physical activity enhances cognitive reserve through mechanisms like increased cerebral blood flow, neurogenesis, and neurotrophic factor upregulation, benefiting individuals regardless of *APOE* genotype.^68^, ^69^ However, studies have reported more pronounced effects in *APOE ε*4 carriers,^14^, ^54^ and the absence of similar findings here could be due to limited power to detect small effects or differences in measurement methods. The differing findings regarding CRUS may also reflect the temporal nature of exposures; CRUS captures earlier life factors, whereas mindfulness and social engagement represent current lifestyle choices, which may interact more dynamically with *APOE ε*4 status in carriers.

### 4.1 Limitations and future directions

Several limitations of this study should be considered. The cross‐sectional design precludes causal conclusions, underscoring the need for longitudinal data to establish the directionality between lifestyle factors, *APOE ε*4 status, and cognitive reserve. Leveraging the ongoing longitudinal data from the HBI could help clarify whether lifestyle activities enhance cognitive reserve or if individuals with higher cognitive reserve are more likely to engage in these activities.

The sample's demographic makeup—primarily White non‐Hispanic (86%), highly educated (mean years = 16.38), and predominantly female (66%)—limits generalizability. A more diverse sample is needed to assess whether the findings apply across different racial, ethnic, and socioeconomic groups. Addressing the underrepresentation of minorities is critical for ensuring that personalized prevention strategies are effective and equitable across all populations, aligning research with diversity, equity, and inclusion principles.

While this study primarily focused on cognitive reserve, brain reserve—the brain's structural capacity, including brain size, neuronal count, and synaptic density^70^—also plays a role in interpreting these findings. Brain reserve complements cognitive reserve by considering the brain's physical robustness, which may interact with cognitive reserve to impact resilience to neuropathology. For *APOE ε*4 carriers, higher brain reserve might enhance resilience to *APOE ε*4‐associated neuropathology and amplify the benefits of lifestyle interventions on cognitive reserve. Conversely, individuals with lower brain reserve may be more susceptible to cognitive decline, even with positive lifestyle factors, due to limited structural capacity to compensate for brain damage.

Our study focused on brain atrophy and WMH as key indicators of brain pathology due to their established links to cognitive decline. However, these measures do not fully capture the spectrum of brain integrity relevant to cognitive reserve. Additional microvascular damage, such as cerebral microbleeds or lacunar infarcts, and broader neurodegenerative markers could provide a more comprehensive view of brain pathology. Recent studies incorporating these markers have developed refined measures of residual cognitive reserve.^71^, ^72^ Thus, our findings of stronger associations between lifestyle factors and cognitive reserve in A*POE ε*4 carriers may reflect differences in both brain and cognitive reserve. This aligns with the view that cognitive and brain reserve jointly contribute to resilience, with brain reserve potentially buffering the impact of genetic risks like *APOE ε*4. Future research should include assessments of brain reserve and a broader range of neuroimaging biomarkers to better understand how these factors interact to influence cognitive health in aging.

Despite the limitations, the study has several strengths. It used a well‐characterized, deeply‐phenotyped cohort. The comprehensive assessment of lifestyle factors using validated scales enhances the reliability of the findings, providing a foundation for future research. The novelty of this study lies in its comprehensive exploration of the interactive effects between *APOE ε*4 status and multiple lifestyle factors on cognitive reserve, particularly emphasizing mindfulness and social engagement—areas that have not been extensively examined in prior research. This is the first study to investigate the moderating effects of mindfulness practices on cognitive outcomes specifically in *APOE ε*4 carriers, providing new insights into personalized, non‐pharmacological strategies for enhancing cognitive resilience in this high‐risk population.

Future research should explore other genetic markers that influence the relationship between lifestyle factors and cognitive outcomes. The *BDNF* Val66Met polymorphism, for instance, is linked to cognitive function and brain plasticity, with the Met allele potentially reducing the cognitive benefits of physical activity and lifestyle interventions.^73^ Variants in genes such as *CLU*, *PICALM*, *CR1*, and *TOMM40* are also associated with Alzheimer's risk and may interact with lifestyle factors to shape cognitive trajectories.^74^, ^75^ Integrating these genetic markers could enhance our understanding of how genetic predispositions modulate lifestyle intervention effectiveness, advancing personalized prevention strategies for cognitive health in aging. Additionally, examining the differential impact of carrying one versus two copies of the *APOE ε*4 allele could provide insights into cognitive decline risk, though our study lacked the power to explore this due to the limited number of *ε*4/*ε*4 carriers (*n* = 2).

## 5 CONCLUSION

In sum, the enhanced susceptibility to cognitive decline in *APOE ε*4 carriers may render the brain more receptive to the beneficial effects of social engagement and mindfulness practices, possibly through stress reduction and anti‐inflammatory pathways. This interaction between genetic predisposition and lifestyle factors highlights the potential for targeted interventions to enhance cognitive reserve, particularly in those at higher genetic risk. These findings underscore the importance of incorporating genetic profiles, such as A*POE ε*4 status, into precision medicine approaches to develop more effective, individualized interventions, potentially enhancing cognitive reserve and slowing neurodegeneration.

## CONFLICT OF INTEREST STATEMENT

The authors declare no conflicts of interest. Author disclosures are available in the Supporting Information.

## CONSENT STATEMENT

All human subjects provided informed consent.

## Supporting information



Supporting Information

ICMJE Disclosure Form
