# Supplementary material for: APOE ε4 carrier status moderates the effect of lifestyle factors on cognitive reserve
Source: Alzheimers Dement. 2024 Oct 11;20(11):8062–73. doi: 10.1002/alz.14304 (PMC11567825; doi:10.1002/alz.14304)
Supplement: Supplementary file 1 — Supporting Information [file ALZ-20-8062-s002.docx]

**Supplementary material**

**Page 2:** List of cognitive tests administered.

**Page 3-4:** Table S1. Description of lifestyle measures

**Page 4-5:** Table S2**.** Sensitivity Analysis of SEM Models for Two-Way Interactions by Age Group (Younger than 65 vs. 65 and Older)

**Page 5:** Table S3. Sensitivity Analysis of SEM Models for Education and Occupation Effects on Cognitive Reserve

**Page 6:** Table S4. SEM Model Analysis of Three-Way Interactions Between Lifestyle Factors, APOE ε4 Status, and Cognitive Status

**Cognitive Tests Administered.**

The cognitive assessments utilized in this study were derived from the National Alzheimer’s Coordinating Center (NACC) Uniform Data Set (UDS: version 3)[1]. The Montreal Cognitive Assessment (MoCA) for global cognitive function[2], the Digit Span tests (forwards and backwards) for auditory attention and working memory [3], the Animal Naming test [4] for semantic fluency, the 32-item Multilingual Naming Test (MINT) for naming and language[5], the Trail Making Test (TMT)[6] for processing speed and executive function, the Number Symbol Coding task for executive function [8], the Clock Drawing Test (on the MoCA) for visuospatial abilities, the Craft Story 21 immediate and delayed recall for verbal episodic memory[7], and the Benson Complex Figure Copy for visuo-constructional ability[8].The Hopkins Verbal Learning Test-Revised (HVLT-R)[9] for verbal memory and the Noise Pareidolia Task (Short Form) for visual perception [10] were also included in the cognitive tests battery administered to HBI participants.

**Table S1.** Description of sub-scales used in the calculation of the Resilience Index.

| **Scale** | **Description** | **Range** |
| --- | --- | --- |
| Cognitive Reserve Unit Scale [11] | Standardized measurement of seven levels of educational and occupational attainment (higher scores indicating greater cognitive reserve) | 0-66 |
| Social Engagement Scale [12] | Measurement of participation in social activities, socialization, and engagement, utilizing a Likert scale (higher scores indicating greater social engagement) | 1-4 |
| Quick Physical Activity Rating [13] | Measurement of intensity, frequency, and duration in 10 categories of physical activities (higher scores indicating greater physical activity) | 0-153 |
| Cognitive & Leisure Activity Scale[12] | Measurement of frequency of participation in 15 categories of cognitively stimulating activities (higher scores indicating greater cognitive activities) | 0-80 |
| Mediterranean-DASH Intervention for Neurodegenerative Delay (MIND) Diet Score Sheet [14] | Measurement (via checklist) of 15 categories of foods with three frequencies of intake (higher scores indicating greater adherence to MIND diet) | 0-15 |
| Applied Mindfulness Process Scale [15] | Measurement of the practice of three aspects of mindfulness: decentering, positive, emotional regulation, and negative emotional regulation (higher scores indicating greater mindfulness) | 0-60 |

| **Table S2** Sensitivity Analysis of SEM Models for Two-Way Interactions by Age Group (Younger than 65 vs. 65 and Older) | | | | | | | | | |
| --- | --- | --- | --- | --- | --- | --- | --- | --- | --- |
|  | |  | |  | |  | |  | |
|  | | β | | **Std. Error** | | **z-value** | | **p-value** | |
| **Young** | |  | |  | |  | |  | |
| **Cognitive Reserve ~** | |  | |  | |  | |  | |
| CRUS | | 0.216 | | 0.191 | | 1.403 | | 0.161 | |
| Social engagement | | -0.053 | | 0.217 | | -0.246 | | 0.806 | |
| Physical Activity | | 0.243 | | 0.245 | | 1.078 | | 0.281 | |
| Cognitive Activity | | 0.181 | | 0.148 | | 1.02 | | 0.308 | |
| MIND diet | | -0.048 | | 0.159 | | -0.304 | | 0.761 | |
| Mindfulness | | -0.225 | | 0.193 | | -1.274 | | 0.203 | |
| Age | | -0.052 | | 0.246 | | -0.389 | | 0.697 | |
| Sex | | -0.039 | | 0.131 | | -0.305 | | 0.760 | |
| APOE e4 | | -0.171 | | 0.234 | | -1.499 | | 0.134 | |
| APOE e4:CRUS | | 0.031 | | 0.354 | | 0.175 | | 0.861 | |
| APOE e4:Social engagement | | 0.548 | | 0.321 | | 2.391 | | 0.017 | |
| APOE e4:Physical Activity | | -0.44 | | 0.355 | | -1.765 | | 0.078 | |
| APOE e4:Cognitive Activity | | -0.218 | | 0.261 | | -1.27 | | 0.204 | |
| APOE e4:MIND diet | | 0.525 | | 0.351 | | 2.419 | | 0.016 | |
| APOE e4: Mindfulness | | 0.18 | | 0.381 | | 0.899 | | 0.369 | |
| **Old** |  | |  | |  | |  | |  |
| **Cognitive Reserve ~** | **β** | | **Std. Error** | | **z-value** | | **p-value** | |  |
| CRUS | 0.415 | | 0.082 | | 4.557 | | <0.001 | |  |
| Social engagement | -0.17 | | 0.093 | | -1.753 | | 0.08 | |  |
| Physical Activity | 0.09 | | 0.08 | | 1.045 | | 0.296 | |  |
| Cognitive Activity | -0.042 | | 0.092 | | -0.473 | | 0.636 | |  |
| MIND diet | -0.088 | | 0.087 | | -0.954 | | 0.34 | |  |
| Mindfulness | 0.024 | | 0.08 | | 0.28 | | 0.779 | |  |
| Age | -0.427 | | 0.113 | | -5.578 | | <0.001 | |  |
| Sex | 0.099 | | 0.073 | | 1.275 | | 0.202 | |  |
| APOE e4 | 0.195 | | 0.173 | | 2.441 | | 0.015 | |  |
| APOE e4:CRUS | -0.141 | | 0.18 | | -1.457 | | 0.145 | |  |
| APOE e4:Social engagement | 0.195 | | 0.157 | | 1.986 | | 0.047 | |  |
| APOE e4:Physical Activity | -0.118 | | 0.201 | | -1.251 | | 0.211 | |  |
| APOE e4:Cognitive Activity | -0.015 | | 0.249 | | -0.148 | | 0.883 | |  |
| APOE e4:MIND diet | -0.021 | | 0.177 | | -0.237 | | 0.813 | |  |
| APOE e4: Mindfulness | 0.211 | | 0.238 | | 1.793 | | 0.073 | |  |

|  |  |  |  |  |  |  |
| --- | --- | --- | --- | --- | --- | --- |
| **Table S3** Sensitivity Analysis of SEM Models for Education and Occupation Effects on Cognitive Reserve | | | | | |  |
|  |  |  |  |  |  |  |
| **Predictor** | **Estimate** | **Std. Error** | **z-value** | **p-value** | **FDR p-adjust** | |
| Occupation | 0.297 | 0.082 | 3.604 | <0.001 | 0.008 |  |
| Social engagement | -0.116 | 0.095 | -1.214 | 0.225 | 0.400 |  |
| Physical Activity | 0.137 | 0.082 | 1.65 | 0.099 | 0.264 |  |
| Cognitive Activity | 0.017 | 0.086 | 0.192 | 0.848 | 0.905 |  |
| MIND diet | -0.075 | 0.085 | -0.869 | 0.385 | 0.573 |  |
| Mindfulness | -0.024 | 0.082 | -0.289 | 0.773 | 0.883 |  |
| Age | -0.34 | 0.068 | -5.004 | <0.001 | 0.008 |  |
| Sex | 0.027 | 0.068 | 0.396 | 0.692 | 0.852 |  |
| APOE e4 | 0.11 | 0.151 | 1.569 | 0.117 | 0.267 |  |
| education | 0.13 | 0.068 | 1.911 | 0.056 | 0.179 |  |
| APOE e4:occupation | -0.071 | 0.157 | -0.852 | 0.394 | 0.573 |  |
| APOE e4:Social engagement | 0.244 | 0.149 | 2.574 | 0.01 | 0.040 |  |
| APOE e4:  Physical Activity | -0.122 | 0.161 | -1.42 | 0.156 | 0.312 |  |
| APOE e4: Cognitive Activity | 0.005 | 0.175 | 0.053 | 0.957 | 0.957 |  |
| APOE e4:MIND diet | 0.061 | 0.154 | 0.745 | 0.456 | 0.608 |  |
| APOE e4:Mindfulness | 0.232 | 0.176 | 2.666 | 0.008 | 0.040 |  |

**Table S4** SEM Model Analysis of Three-Way Interactions Between Lifestyle Factors, APOE ε4 Status, and Cognitive Status

| **Cognitive reserve ~** | **β** | **Std. Error** | **z-value** | | **p-value** | **p-adjust** |
| --- | --- | --- | --- | --- | --- | --- |
|  |  |  |  |  | |  |
| CRUS | 0.275 | 0.059 | 4.641 | < .001 | | 0.0073 |
| Social engagement | -0.083 | 0.062 | -1.34 | 0.18 | | 0.3148 |
| Physical Activity | 0.103 | 0.062 | 1.663 | 0.096 | | 0.192 |
| Cognitive Activity | -0.044 | 0.064 | -0.693 | 0.488 | | 0.6985 |
| MIND diet | -0.081 | 0.062 | -1.322 | 0.186 | | 0.3148 |
| Mindfulness | -0.042 | 0.063 | -0.662 | 0.508 | | 0.6985 |
| Age | -0.27 | 0.064 | -4.245 | < .001 | | 0.0073 |
| Sex | 0.011 | 0.059 | 0.179 | 0.858 | | 0.8989 |
| APOE e4 | 0.155 | 0.143 | 2.372 | 0.018 | | 0.066 |
| cognitive status | -0.337 | 0.067 | -5.4 | < .001 | | 0.0073 |
| APOE e4:CRUS | -0.335 | 0.277 | -2.38 | 0.017 | | 0.066 |
| APOE e4:Social engagement | 0.048 | 0.242 | 0.317 | 0.751 | | 0.8701 |
| APOE e4:Physical Activity | -0.407 | 0.292 | -2.624 | 0.009 | | 0.0495 |
| APOE e4:Cognitive Activity | 0.237 | 0.277 | 1.763 | 0.078 | | 0.1716 |
| APOE e4:MIND diet | 0.035 | 0.253 | 0.265 | 0.791 | | 0.8701 |
| APOE e4:Mindfulness | 0.204 | 0.351 | 1.19 | 0.234 | | 0.3677 |
| APOE e4:CRUS:cognitive status | 0.264 | 0.149 | 1.93 | 0.054 | | 0.1697 |
| APOE e4:Social engagement: cognitive status | 0.093 | 0.118 | 0.53 | 0.596 | | 0.7713 |
| APOE e4:Physical Activity: cognitive status | 0.307 | 0.153 | 1.801 | 0.072 | | 0.1716 |
| APOE e4:Cognitive Activity: cognitive status | -0.24 | 0.161 | -1.808 | 0.071 | | 0.1716 |
| APOE e4:MIND diet: cognitive status | 0.044 | 0.138 | 0.3 | 0.764 | | 0.8701 |
| APOE e4:Mindfulness:cognitive status | -0.012 | 0.157 | -0.065 | 0.948 | | 0.948 |
| Model R-Square | 0.489 |  |  |  | |  |
| Chi-square | 362.279 |  |  |  | |  |
| df | 96 |  |  |  | |  |

References

1. Weintraub S, Besser L, Dodge HH, et al. Version 3 of the Alzheimer Disease Centers’ neuropsychological test battery in the Uniform Data Set (UDS). Alzheimer Disease & Associated Disorders 2018;**32**(1):10-17.

2. Nasreddine ZS, Phillips N, Chertkow H. Normative data for the Montreal Cognitive Assessment (MoCA) in a population-based sample. 2012.

3. Wechsler D. Wechsler memory scale-revised. Psychological Corporation 1987.

4. Campagna F, Montagnese S, Ridola L, et al. The animal naming test: an easy tool for the assessment of hepatic encephalopathy. Hepatology 2017;**66**(1):198-208.

5. Ivanova I, Salmon DP, Gollan TH. The multilingual naming test in Alzheimer's disease: clues to the origin of naming impairments. Journal of the International neuropsychological Society 2013;**19**(3):272-83.

6. Battery AIT. Manual of directions and scoring: Washington, DC: War Department, Adjutant General’s Office, 1944.

7. Craft S, Newcomer J, Kanne S, et al. Memory improvement following induced hyperinsulinemia in Alzheimer's disease. Neurobiology of aging 1996;**17**(1):123-30.

8. Possin KL, Laluz VR, Alcantar OZ, Miller BL, Kramer JH. Distinct neuroanatomical substrates and cognitive mechanisms of figure copy performance in Alzheimer's disease and behavioral variant frontotemporal dementia. Neuropsychologia 2011;**49**(1):43-48.

9. Benedict RH, Schretlen D, Groninger L, Brandt J. Hopkins Verbal Learning Test–Revised: Normative data and analysis of inter-form and test-retest reliability. The Clinical Neuropsychologist 1998;**12**(1):43-55.

10. Turner TH, Rodriguez‐Porcel F. Utility of the 20‐Item Noise Pareidolia Task (NPT‐20) for Assessing Visuoperceptual Disturbances Associated with Complex Visual Hallucinations in Parkinson's Disease. Movement Disorders Clinical Practice 2023;**10**(2):269-73.

11. Joshi MS, Galvin JE. Cognitive resilience in brain health and dementia research. Journal of Alzheimer's Disease 2022;**90**(2):461-73.

12. Galvin JE, Tolea MI, Chrisphonte S. The Cognitive & Leisure Activity Scale (CLAS): A new measure to quantify cognitive activities in older adults with and without cognitive impairment. Alzheimer's & dementia: translational research & clinical interventions 2021;**7**(1):e12134.

13. Galvin JE, Tolea MI, Rosenfeld A, Chrisphonte S. The Quick Physical Activity Rating (QPAR) scale: A brief assessment of physical activity in older adults with and without cognitive impairment. PLoS One 2020;**15**(10):e0241641.

14. Talegawkar SA, Jin Y, Simonsick EM, Tucker KL, Ferrucci L, Tanaka T. The Mediterranean-DASH Intervention for Neurodegenerative Delay (MIND) diet is associated with physical function and grip strength in older men and women. The American journal of clinical nutrition 2022;**115**(3):625-32.

15. Li MJ, Black DS, Garland EL. The Applied Mindfulness Process Scale (AMPS): A process measure for evaluating mindfulness-based interventions. Personality and individual differences 2016;**93**:6-15.
